# Investigation of outcomes following transcatheter edge to edge repair of the mitral valve versus medical management alone in patients with cardiogenic shock and mitral regurgitation

**DOI:** 10.1016/j.ahjo.2024.100430

**Published:** 2024-07-29

**Authors:** Caleb J. Chiang, Mina Kerolos, Michael Sunnaa, Sushant Koirala, Joseph Eid, Ethan M. Ritz, Laith A. Derbas, Fareed Moses Collado, Tisha M. Suboc, Clifford J. Kavinsky, Hussam S. Suradi

**Affiliations:** a Division of Cardiology, University of Minnesota, Minneapolis, MN, United States of America; b Department of Internal Medicine, Rush University Medical Center, Chicago, IL, United States of America; c Rush Bioinformatics and Biostatistics Core, Rush University Medical Center, Chicago, IL, United States of America; d Division of Cardiology, Rush University Medical Center, Chicago, IL, United States of America

**Keywords:** Mitraclip, Mitral valve, Severe mitral regurgitation, Transcatheter edge to edge repair, Cardiogenic shock

## Abstract

**Study objective:**

Assessing if Transcatheter Edge to Edge Repair (TEER) with Mitraclip™ in patients with moderate to severe mitral regurgitation (MR) and cardiogenic shock (CS) improves outcomes compared to medical management alone.

**Design:**

A single-center, retrospective study was performed in an urban tertiary referral center.

**Setting:**

Rush University Medical Center, United States.

**Participants:**

Adult patients presenting with CS and moderate to severe MR between 2012 and 2021 were included.

**Interventions:**

Undergoing Mitral TEER with Mitraclip versus medical management alone.

**Main outcome measures:**

Major adverse cardiovascular events (MACE) defined as cardiovascular death, heart failure admission, stroke, and myocardial infarction assessed at 30 days, 6 months, and 1 year. The secondary outcome was a change in New York Heart Association (NYHA) classification at 30 days and 6 months.

**Results:**

There were 28 patients included in the medical management and 33 in the mitral valve TEER groups. There was a decreased MACE in the intervention group at 30 days (24.2 % vs. 46.4 %, *p* ≤0.001) and 6 months (27 % vs. 75 %, *p* = 0.002), though not at 1 year (29.4 % vs. 41.7 %, *p* = 0.42). At 30 days, more patients in the mitral valve TEER group improved to NYHA classes I/II compared to medical management alone (10 [35.7 %] vs. 16 [50 %], *p* = 0.043). There were no differences in NYHA classes I/II at 6 months (7 [43.7 %] vs. 13 [54.2 %], *p* = 0.63).

**Conclusion:**

Mitral valve TEER using the Mitraclip™ system improves mid-term cardiovascular compared to medical management alone in patients with CS but does not improve mortality.

## 1 Introduction

Cardiogenic shock (CS) is a serious medical condition that results in hemodynamic instability, hypoxia, and end-organ damage and is associated with high mortality and morbidity [[1], [2], [3]].

Myocardial infarction (MI) is the most common cause of CS [4], although significant mitral regurgitation (MR) may be present in up to 10 % of patients with CS [5]. Moderate to severe MR may result in a significant reduction in cardiac output (CO), further worsening hemodynamics in the setting of CS, and is an independent risk factor for mortality [6,7]. In addition, such patients are often at high surgical risk, and medical management alone of MR in CS is associated with significantly increased mortality [8].

Transcatheter Edge to Edge Repair (TEER) of the mitral valve is a viable intervention for MR and carries a 2 A recommendation in patients with severely symptomatic primary MR at high surgical risk^9^. Landmark trials previously established the efficacy and safety of the MitraClip™ device (Abbott, IL, USA) in patients with heart failure (HF) and chronic MR [10,11]. Greater interest has recently been directed toward using mitral valve TEER in CS. A growing body of evidence suggests that mitral valve TEER may be a viable option for moderate to severe MR in CS patients who are not surgical candidates [4,12]. We hypothesized that mitral valve TEER compared to medical management alone would improve outcomes in patients with moderate to severe mitral regurgitation and CS.

## 2 Materials and methods

### 2.1 Study design

This single-center, retrospective study was performed in a large, urban, tertiary referral center. A trained clinician collected demographic information, echocardiographic details, and procedural data using departmental procedural databases and the hospital's electronic medical record (Epic®, Verona, WI). This study was approved by the Rush University Medical Center Institutional Review Board (#20062201).

Adult patients (18 years or older) presenting with CS and moderate to severe MR between January 2012 and December 2021 were included. Moderate to severe MR was defined as grades 3+ or 4+ MR identified via echocardiography. CS was defined as a sustained systolic blood pressure < 90 mmHg for at least 1 h, use of inotropes, vasopressors, or mechanical circulatory support (MCS, defined as Impella, Intra-aortic balloon pump, or ventricular assist device), and clinical and laboratory findings of end-organ damage. Mitral valve TEER selection was based on a multidisciplinary heart team approach after patients were deemed prohibitively high-risk surgical candidates. Patients in the control group were all consecutive patients who had CS and MR based on chart review. Exclusion criteria included a history of surgical or transcatheter mitral valve repair, absence of clip deployment, MR grade < 3+, or absence of intensive care unit (ICU) admission.

Outcomes were compared between the control group (medical management alone) and the intervention group (TEER with Mitraclip device™ in addition to medical management). The primary outcome was major adverse cardiovascular events (MACE), defined as cardiovascular death, heart failure admission, stroke, and myocardial infarction at 30 days, 6 months, and 1 year. The secondary outcome was a New York Heart Association (NYHA) classification change. In the intervention group, time-to-event data was recorded from the procedure date to the date of the event, while the control group used the date of discharge to the date of the event. In addition, time to heart failure readmission was also compared from the time of discharge to the event in both arms.

### 2.2 Statistical analysis

When appropriate, categorical outcomes were compared with chi-squared tests or Fisher's exact test. *t*-Tests were used when evaluating continuous outcomes. For ordinal data (MR grade, NYHA), Wilcoxon tests were used. All analyses were conducted using R© version 4.2.2.

## 3 Results

A total of 472 records were obtained using associated ICD-10 codes and departmental procedural databases. After removing exclusions, the final cohort consisted of 28 patients in the medical management alone group and 33 in the mitral valve TEER group. Baseline characteristics for both groups are included in Table 1. The two groups' baseline characteristics were mostly comparable. The control group had a significantly higher Black or African American population (82.1 % vs. 42.4 %, *p* = 0.009), while the intervention group had a higher percentage of white patients (39.4 % vs. 14.3 %, p = 0.009). The control group had a lower ejective fraction than the intervention group (22 % vs. 36 %, *P* = 0.001). A significantly higher number of patients in the control group had a history of ventricular tachycardia (28.6 % vs. 9.1 %, *p* = 0.049). More patients in the control group had been on guideline-directed medical therapy and treated with renin-angiotensin inhibition compared to the intervention group (60.7 % vs. 33.4 %, *p* = 0.032), beta blockers (82.1 % vs. 66.7 %, *p* = 0.0171), aldosterone antagonist (42.9 % vs. 36.4 %, *p* = 0.171). Although treatment with beta-blockers and aldosterone antagonists was numerically higher in the control group, this was not statistically significant. There were no significant differences in baseline NYHA class, Society of Thoracic Surgeons (STS) score, or etiology of cardiomyopathy. The loss to follow up rate in the medical management alone group was 33.3 % in the 6-month time period, and 25.0 % in 1 year. Whereas the loss to follow up rate in the TEER group was 7.1 % in the 6-month time period and 26 % in the 1 year time period.

**Table 1:** Baseline characteristics.

	Control (*n* = 28)	Intervention (*n* = 33)	*P-value*
Age (years, ±SD)	62.14 (18.18)	67.30 (12.10)	0.191
Gender (%)			0.754
Female	15(53.6)	19(57.6)	
Male	13(46.4)	14(42.4)	
Race (%)			0.009
Asian	0	2 (6.1)	
Black or African American	23 (82.1)	14 (42.4)	
White	4 (14.3)	13 (39.4)	
Other	1 (3.6)	4 (12.1)	
Hispanic/Latino (%)	1 (3.6)	4 (12.1)	0.363
BMI (mean, ±SD)	26.94 (8.34)	27.37 (7.00)	0.828
Baseline NYHA (%)			0.373
NYHA 2	1 (3.6)	0 (0.0)	
NYHA 3	16 (57.1)	16 (48.5)	
NYHA 4	11 (39.3)	17 (51.5)	
BNP (mean, ±SD)	2724.00 (2368.78)	2120.79 (1762.08)	0.286
STS (mean ± SD)	7.52 (5.34)	10.28 (8.13)	0.13
Cardiomyopathy (%)			0.168
Ischemic	5 (17.9)	9 (27.3)	
Non-ischemic	23 (82.1)	20 (60.6)	
Mixed	0	1 (3.0)	
No prior cardiomyopathy	0	3 (9.1)	
Diabetes (%)	10 (35.7)	13 (39.4)	0.768
Dyslipidemia (%)	14 (50.0)	18 (54.5)	0.723
Hypertension (%)	27 (96.4)	26 (78.8)	0.06
Stroke/TIA (%)	7 (25.0)	4 (12.1)	0.192
Chronic lung disease (%)	14 (50.0)	13 (39.4)	0.406
Pulmonary hypertension (%)	9 (64.3)	9 (69.2)	1
Chronic kidney disease ≥ stage 2	11 (39.3)	17 (51.5)	0.34
Afib/flutter(%)	13 (46.4)	19 (57.6)	0.385
History of ventricular tachycardia (%)	8 (28.6)	3 (9.1)	0.049
CAD (%)	10 (35.7)	17 (51.5)	0.216
History of MI (%)	5 (17.9)	11 (33.3)	0.171
Acute MI (%)	1 (3.6)	2 (6 %)	1
Prior PCI (%)	5 (17.9)	7 (21.2)	0.743
Prior CABG (%)	1 (3.6)	7 (21.2)	0.06
PVD (%)	2 (7.1)	0	0.207
PPM/ICD (%)	11 (39.3)	12 (36.4)	0.814
On home inotropes	1 (3.6)	4 (12.1)	0.363
Aspirin (%)	18 (64.3)	19 (57.6)	0.593
P2Y12 inhibitor (%)	0	4 (12.1)	0.118
Prior therapeutic AC (%)	12 (42.9)	17 (51.5)	0.5
Statin (%)	14 (50.0)	23 (69.7)	0.117
ACEI/ARB/ARNI (%)	17 (60.7)	11 (33.3)	0.032
Beta-blocker (%)	23 (82.1)	22 (66.7)	0.171
Hydralazine (%)	3 (10.7)	6 (18.2)	0.488
Aldosterone antagonist (%)	12 (42.9)	12 (36.4)	0.605

*Abbreviations*: AC, anticoagulation; ACEI, angiotensin-converting enzyme inhibitor; Afib/flutter, atrial fibrillation, atrial flutter; ARB, angiotensin II receptor blocker; ARNI, angiotensin receptor/neprilysin inhibitor; BMI, body mass index; BNP, brain natriuretic peptide; CABG, coronary artery bypass graft; CAD, coronary artery disease; CKD, chronic kidney disease; ICD, implantable cardioverter defibrillator; MI, myocardial infarction; NYHA, New York Heart Association; PCI, percutaneous coronary intervention; PVD, peripheral vascular disease; SD, standard deviation; STS, Society of Thoracic Surgeons; TIA, transient ischemic attack.

Table 2 summarizes key baseline echocardiographic data for the cohort. The mean left ventricular ejection fraction (LVEF) was significantly lower in the control group compared to the intervention group (22.43 % vs. 36.42 %, *p* = 0.001). Additionally, the control group had a larger mean left ventricular end-diastolic diameter than the intervention group (6.97 cm vs. 5.86 cm, *p* = 0.001). More patients in the intervention group had reported degenerative mitral regurgitation (DMR, 45.5 % vs 7.1 %, *P* < 0.001). In contrast, the control group had a higher rate of functional mitral regurgitation (FMR, 85.7 % vs. 27.3 %, P < 0.001). There were no differences in the incidence of MR grades 3+/4+ in the cohorts. Rates of concomitant TR and AS were comparable between the two groups. RV function and tricuspid annular plane systolic excursion (TAPSE) were similar between the control and intervention groups. The intervention group had a higher baseline pulmonary artery systolic pressure (PASP) compared to the control group (*p* = 0.003).

**Table 2:** Baseline echocardiographic data.

	Control (n = 28)	Intervention (n = 33)	*P-value*
LVEF (%, mean, ±SD)	22.43 (8.37)	36.42 (20.16)	0.001
LVEDD (cm, mean ± SD)	6.97 (1.18)	5.86 (1.09)	0.001
MR grade (%)			0.814
3+	11 (39.3)	12 (36.4)	
4+	17 (60.7)	21 (63.6)	
MR etiology (%)			<0.001
DMR	2 (7.1)	15 (45.5)	
FMR	24 (85.7)	9 (27.3)	
Mixed	2 (7.1)	9 (27.3)	
TR grade (%)			0.105
0	4 (14.3)	1 (3.0)	
1	9 (32.1)	8 (24.2)	
2	7 (25.0)	12 (36.4)	
3	5 (17.9)	2 (6.1)	
4	3 (10.7)	10 (30.3)	
AS (%)	2 (7.1)	4 (12.1)	0.678
Trace/Trivial	0	4 (12.1)	0.058
Mild	1 (3.6)	0 (0.0)	
Severe	1 (3.6)	0 (0.0)	
TAPSE (cm; mean ± SD)	1.80 (0.64)	1.58 (0.50)	0.397
PASP (mmHg; mean ± SD)	48.73 (13.31)	61.65 (17.64)	0.003
RV function (%)			0.955
Normal	10 (35.7)	11 (33.3)	
Mild dysfunction	5 (17.9)	5 (15.2)	
Moderate dysfunction	11 (39.3)	15 (45.5)	
Severe dysfunction	2 (7.1)	2 (6.1)	

*Abbreviations*: AS, aortic stenosis; DMR, degenerative mitral regurgitation; FMR, functional mitral regurgitation; LVEDD, left ventricular end-diastolic diameter; LVEF, left ventricular ejection fraction; MR, mitral regurgitation; PASP, pulmonary arterial systolic pressure; RV, right ventricle; TAPSE, tricuspid annular plane systolic excursion; TR, tricuspid regurgitation.

### 3.1 Primary and secondary outcomes

Table 3 summarizes the outcomes in the two groups. Regarding the primary outcome, there was a decreased MACE in the intervention group at 30 days (24.2 % vs. 46.4 %, *p* ≤0.001) and 6 months (27 % vs. 75 %, *p* = 0.002), though not at 1 year (29.4 % vs. 41.7 %, *p* = 0.42). There were no differences in cardiovascular deaths at any time point. One patient in the intervention group experienced a myocardial infarction (*p* ≤0.001). No patients experienced strokes. There were significantly fewer heart failure admissions in the intervention group at 6 months (12/16 (75 %) vs. 6/26 (23.1 %), *p* ≤0.001).

**Table 3:** Outcomes.

In-hospital	Control (n = 28)	Intervention (n = 33)	*P*-value
CV death	2(7.1)	3(9.1)	
Stroke	0	0	
MI	0	0	
All-cause mortality	2(7.1)	3(9.1)	

30 day outcomes			
MACE	13 (46.4)	8 (24.2)	<0.001
CV death	8 (28.6)	4 (12.1)	0.107
HF admission	7 (25)	4 (12.1)	0.19
Stroke	0	0	NA
MI	0	1 (3.0)	<0.001
All-cause mortality	8 (28.6)	5 (15.2)	0.202


6 month outcomes	Control (*n* = 16)	Intervention (*n* = 26)	*P*-value
MACE	12 (75)	7 (27)	0.002
CV death	0	3 (11.5)	0.275
HF admission	12 (75)	6 (23.1)	<0.001
Stroke	0	0	NA
MI	0	0	NA
All-cause mortality	0	3 (11.5)	0.275


1 year outcomes	Control (*n* = 12)	Intervention (*n* = 17)	*P*-value
MACE	5 (41.7)	5 (29.4)	0.42
CV death	1 (8.3)	1 (5.9)	1
HF admission	4 (33.3)	5 (29.4)	0.82
Stroke	0	0	NA
MI	0	0	NA
All-cause mortality	1 (8.3)	1 (5.9)	1

*Abbreviations*: CV, cardiovascular; HF, heart failure; MACE, major adverse cardiovascular events; MI, myocardial infarction.

**Fig. 1:**
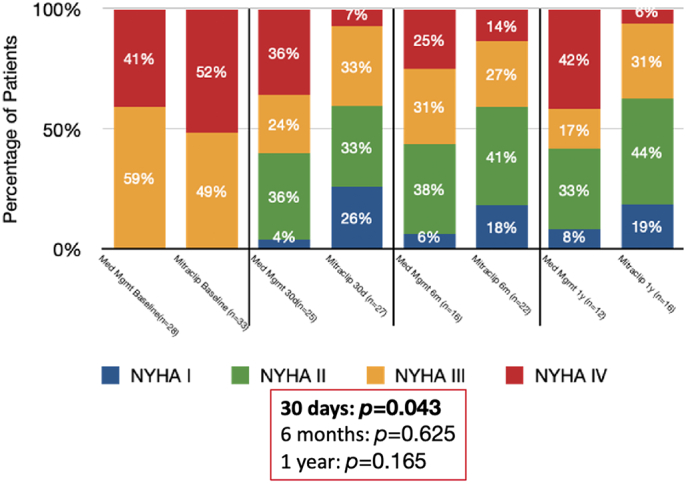
Change in NYHA classification.

Fig. 1 demonstrates changes in NYHA classification in both cohorts at baseline, 30 days, 6 months, and 1 year. At baseline, most patients in both cohorts were NYHA classes III/IV without significant differences between the two groups. At 30 days, there were significant improvements in NYHA classification and greater improvement in the intervention group compared to control (*p* = 0.043). There were sustained improvements in the NYHA class at 6 months and 1 year, though without significant differences between the two groups (*p* = 0.625, *p* = 0.165, respectively).

### 3.2 Procedural and hemodynamic data

Table 4, Table 5 include the cohorts' hemodynamic and procedural data and outcomes. The mean number of Mitraclips™ devices deployed during their procedures was 1.45 (±0.67). There were no differences in inotrope use or duration of use. Patients undergoing mitral valve TEER were more likely to receive vasopressor support (*p* = 0.003) and MCS (*p* = 0.005) compared to the control group. Before mitral TEER, 48 % of patients required an intra-aortic balloon pump. There were no differences in the intubation rate (*p* = 0.072), though the control group was more likely to have a longer duration of intubation (172 h vs. 32 h, *p* = 0.013). Patients receiving Mitraclip™ devices were more likely to have right heart catheterizations for pressure monitoring (*p* = 0.004). At baseline, there was no difference in cardiac output (CO) or cardiac index (CI). Following the intervention, there were significant improvements in cardiac output (CO; *p* = 0.001) and cardiac index (CI; *p* = 0.001) compared to the control group (Table 6).

**Table 4:** Hemodynamic data.

	Control (n = 28)	Intervention (n = 33)	*P*-value
SCAI shock stage			
B/C (%)	20 (71.5)	29 (87.8)	0.113
D/E (%)	8 (28.5)	4 (12.2)	0.113
Lactate (±SD)	3.09 (2.87)	2.25 (1.03)	0.15
Inotrope use (%)	21 (75)	22 (66.7)	0.636
Total time on inotropes (hr, ±SD)	147.67 (155.89)	214.82 (190.33)	0.214
Vasopressor use (%)	7 (25)	21 (63.6)	0.003
Total time on vasopressors (hr, ±SD)	105 (63.16)	75.26 (126.95)	0.551
MCS (%)	4 (14.3)	16 (48.5)	0.005
IABP (%)	3 (10.7)	16 (48.5)	0.005
Impella device (%)	1 (0.04)	0	
Total time on MCS (hr, ±SD)	153.5 (165.40)	101.44 (61.52)	0.303
Intubation (%)	4 (15.4)	12 (36.4)	0.072
Time intubated (hr, ±SD)	171.50 (176.71)	31.96 (23.65)	0.013
Right heart catheterization (%)	18 (64.3)	31 (93.9)	0.004
Initial mean RA pressure (mmHg, ±SD)	14.39 (7.65)	14.82 (7.56)	0.851
Initial mean PA pressure (mmHg, ±SD)	36.22 (7.76)	35.54 (10.36)	0.811
Initial mean PCWP pressure (mmHg, ±SD)	28.90 (13.65)	23.24 (6.88)	0.132
Initial CO (L/min, ±SD)	2.99 (1.06)	5.17 (4.48)	0.059
Initial CI (L/min/m^2^, ±SD)	1.65 (0.680)	2.08 (0.63)	0.052
Initial LA pressure (mmHg, ±SD)	–	22.6 (9.8)	
Final mean RA pressure (mmHg, ±SD)	10.21 (4.74)	13.86 (7.25)	0.105
Final mean PA pressure (mmHg, ±SD)	32.43 (9.99)	32.21 (8.46)	0.946
Final mean PCWP pressure (mmHg, ±SD)	17.75 (8.77)	18.20 (9.04)	0.942
Final CO (L/min, ±SD)	4.02 (1.30)	6.06 (1.30)	<0.001
Final CI (L/min/m^2^, ±SD)	2.23 (0.62)	3.24 (0.5)	<0.001
Final LA pressure (mmHg, ±SD)	–	19.3 (8.5)	

*Abbreviations*: CI = cardiac index; CO = cardiac output; IABP = intraaortic balloon pump; MCS = mechanical circulatory support; PA = pulmonary artery; PCWP = pulmonary capillary wedge pressure; RA = right atrium; LA:Left atrium.

**Table 5:** Procedural data.

	Intervention (n = 33)
Number of clips deployed (±SD)	1.45 (0.67)
Tricuspid valve clip	2 (6.1)
IABP use	16 (48.5)

**Table 6:** Follow-up echocardiographic data.

	Control	Intervention	*P*-value
LVEF (%, ±SD)			
Baseline	22.43 (8.37)	36.42 (20.16)	0.001
30 days	25.88 (13.03)	33.97 (19.76)	0.282
6 months	22.80 (12.69)	29.08 (19.00)	0.383
1 year	32.00 (16.74)	31.33 (17.76)	0.934

PASP (mmHg, ±SD)			
Baseline	48.73 (13.31)	61.65 (17.64)	0.003
30 days	40.80 (12.26)	53.55 (16.19)	0.112
6 months	58.71 (5.28)	44.78 (10.91)	0.008
1 year	44.40 (6.66)	42.00 (13.29)	0.732

MR grade baseline			
3–4+ (%)	28 (100)	33 (100)	1
30 days			0.007
0–2+ (%)	5 (62.5)	27 (87.1)	
3–4+ (%)	3 (73.5)	4 (12.9)	
6 months			0.042
0–2+ (%)	3 (30)	10 (83.4)	
3–4+ (%)	7 (70)	2 (16.6)	
1 year			0.028
0–2+ (%)	5 (62.5)	11 (100)	
3–4+ (%)	3 (37.5)	0	

*Abbreviations*: LVEF, left ventricular ejection fraction; MR, mitral regurgitation; PASP, pulmonary arterial systolic pressure.

### 3.3 Follow-up echocardiographic data

**Fig. 2:**
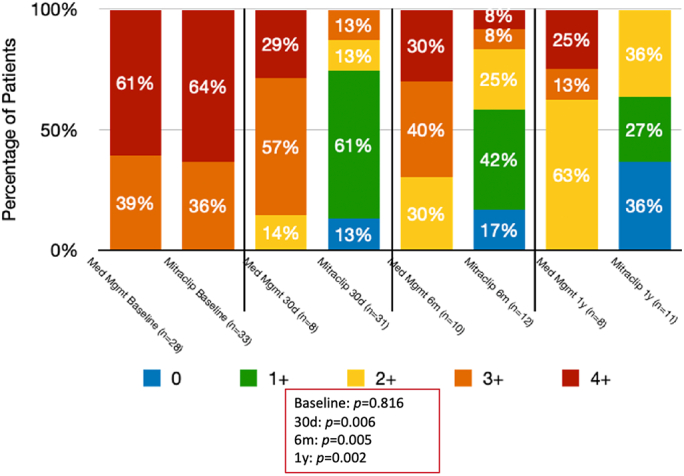
Change in mitral regurgitation grade.

Table 5 includes comparisons of the cohorts' echocardiographic data. There were no changes in LVEF between the control and intervention groups during the duration of follow-up. As previously discussed, PASP was higher in the intervention group at baseline. Following TEER, the PASP decreased over the duration of follow-up, while the control group's PASP remained unchanged. As expected, the intervention group's MR grade significantly improved following mitral valve TEER (Fig. 2). For example, TEER patients had grades 3 and 4 MR, which was reduced to 13 % by 30 days, 16 % by 6 months, and 0 % by 1 year in surviving patients. Compared to the control group, 86 % had grade 3 and 4 MR by 30 days, 70 % by 6 months, and 38 % in one year.

## 4 Discussion

Compared to medical management alone, mitral valve TEER is associated with significant improvements in MACE and functional assessments in patients with CS. Mitral valve TEER also resulted in significant improvements in MR grade, PASP, CO, and CI following this procedure.

Standard medical management of concurrent CS and MR involves inotropic and blood pressure support, MCS device placement, or revascularization. In the setting of severe MR, these solely provide supportive treatment and do not remedy the underlying valvular pathology and progressive nature of heart failure. Prior management of MR in the setting of CS has previously been difficult, as the patients' critical illness, multiple comorbidities, and hemodynamic instability often preclude surgical revision [13,14]. Mitral valve TEER provides a less invasive modality of mitral regurgitation repair. This procedure within CS management has increased over the recent years, with a recent multi-center trial demonstrating 1-year mortality benefit following this procedure [12].

Our study demonstrates significant MACE benefits of mitral valve TEER at 6 months, although not at 30 days or 1 year. Notably, there was not a mortality benefit noted at any of the time periods. This differs from Tang et al., a larger study that noted improvements in mortality through 1 year [12]. All-cause mortality was not statistically different between the control and intervention groups (2 [7.1 %] vs. 3 [9.1 %]). This is substantially lower than reported by Tang et al. (35.4 % vs. 24.8 %) [10], though similar to previous work examining Mitraclip™ use in CS [5,15,16]. We suspect that a number of factors may have contributed to the lower mortality rates seen. Both cohorts were of a relatively small sample size. Most patients had less severe shock (SCAI stage B/C, 71.5 % control, 87.7 % intervention). There is also likely a component of selection bias toward patients who would survive the procedure. As stated earlier, the selection for mitral valve TEER was based on a combined heart team approach and mitral valve anatomy. There were more DMR patients in the TEER group (45.5 % vs. 7.1 %) as opposed to more FMR patients in the control group (85.7 % vs. 27.3 %). Although typically more FMR patients present with shock, shock is a well-established complication in FMR [5].

Compared to controls, there was a significantly decreased rate of HF readmission in the intervention group at 6 months. Mitral valve TEER has previously been shown to reduce the incidence of HF readmission following successful device placement compared to control [10]. However, despite mitral valve TEER, heart failure readmission has remained moderately elevated, with several studies citing a rate of up to 50 % within their follow-up periods [5,10,11]. Our study only demonstrated differences in HF admission at 6 months. This differs from the COAPT trial, which detected differences in HF admissions as early as 30 days when compared to medical management alone [8]. Small sample size again may have contributed to the lack of significant difference at 30 days. However, patients with CS are a far more ill population than compared to the COAPT trial; thus, it is not surprising to have higher proportions of the patients admitted for heart failure exacerbations. Rather, Mitraclip™ may provide a long-term benefit in this area. The authors suspect the lack of difference at one year was due to a significant loss of follow-up in both groups, as previously discussed.

Our data also indicated improvement in symptom severity following Mitraclip™ device placement based on the NYHA classification. There were statistically significant improvements in the NYHA class at 30 days, with sustained improvements at 6 months and 1 year, though without significant differences. Prior research has noted NYHA class improvement compared to medical management, although there are mixed results [8,9]. This study's results are similar to prior research examining outcomes of TEER in CS [17,18]. While our study indicates initial functional status improvement following Mitraclip™, further larger studies are likely necessary to fully elucidate longer-term benefits.

Previous studies showed improvements in left heart volumes and pressures and pulmonary pressures [[19], [20], [21]]. These studies noted improvement in MR, resulting in decreased vascular congestion and LV diastolic volumes, thus reducing LV preload. Similarly, our intervention group sustained a small, acute drop in LVEF following mitral valve TEER (36.42 % to 33.97 %). This may be a result of a reduction in regurgitant volume through the low impedance left atrial pathway, driving volume systemically and acutely increasing the LV afterload. Additionally, the LV end-diastolic volume (LVEDV) may be reduced following mitral valve TEER. However, an increase in forward stroke volume may in part explain the increases in CO and CI seen in our cohort following the procedure, thereby compensating for the mild decrease in LVEF. However, the results may be difficult to interpret given the intervention group frequently required MCS and inotropic support. Moreover, invasive pressure monitoring may have been obtained hours to days following the procedure; thus, medical and volume optimization may have contributed to the observed improvement.

Interestingly, there were racial differences between the intervention and the control group. The majority of the control group consisted of Black individuals compared to the intervention group (82.1 % vs. 42.4 %). Tang et al. also reported high percentages of white patients (82.8 %) in the mitral valve TEER group but with similar percentages of Whites in the control group (77.3 %) [12]. Prior work has made similar observations of racial disparities in the past [22,23]. Though not elucidated within this study, the large differences in utilization are likely multifactorial and may relate to access to medical care, perceptions of invasive therapies, and or systemic biases in patient selection. Further focused research is needed to fully appreciate the complexities of this pervasive issue.

### 4.1 Study limitations

This study has several limitations. First, its retrospective design is observational in nature and susceptible to biases. Second, there were a limited number of patients in both study arms, and controlling for the groups was limited. Thirdly, other variables such, as discharge medication differences between groups, were not investigated nor controlled for. Likewise, missing data and loss of follow-up further limit generalizability of the results.

## 5 Conclusions

Mitral valve TEER using the Mitraclip™ system improves short and mid-term cardiovascular outcomes compared to medical management alone in patients with CS but does not improve mortality.

## Ethics statement

Institutional Review Board Protocol was approved according to institution guidelines. No informed consent was obtained as this was a retrospective study.

## CRediT authorship contribution statement

**Caleb J. Chiang:** Writing – original draft, Project administration, Methodology, Conceptualization. **Mina Kerolos:** Writing – review & editing, Project administration, Investigation, Conceptualization. **Michael Sunnaa:** Writing – review & editing, Project administration. **Sushant Koirala:** Writing – review & editing. **Joseph Eid:** Writing – review & editing, Investigation. **Ethan M. Ritz:** Formal analysis. **Laith A. Derbas:** Writing – review & editing. **Fareed Moses Collado:** Writing – review & editing. **Tisha M. Suboc:** Writing – review & editing. **Clifford J. Kavinsky:** Writing – review & editing. **Hussam S. Suradi:** Supervision, Methodology.

## Declaration of competing interest

The authors declare that they have no known competing financial interests or personal relationships that could have appeared to influence the work reported in this paper.
